# The influence and mechanistic action of sperm DNA fragmentation index on the outcomes of assisted reproduction technology

**DOI:** 10.1515/biol-2022-0597

**Published:** 2023-05-19

**Authors:** Hui Zhang, Fei-Yue Zhu, Xiao-Juan He, Shi-Huan Tang, Ting Long, Lu Peng, Hong-Mei Zhang, Zong-Zhi Zou, Zhu Xiong, Xian-Ping Zhang

**Affiliations:** Department of Reproductive Medicine Center, Loudi Affiliated Hospital, Hengyang Medical School, University of South China, Loudi, 417000, China; Department of Hematology, Loudi Affiliated Hospital, Hengyang Medical School, University of South China, Loudi, 417000, China; Department of Nephropathy Endocrinology, The Second Affiliated Hospital, Hunan University of Chinese Medicine, Changsha, 410000, China; Department of Pediatric Orthopedics, Shenzhen Children’s Hospital of China Medical University, Guangdong, 518034, China

**Keywords:** DNA fragmentation index, reactive oxygen species, apoptosis, *in vitro* fertilization and embryo transfer/intracytoplasmic sperm injection, clinical pregnancy

## Abstract

We investigated the influence of DNA fragmentation index (DFI) on *in vitro* fertilization (IVF), embryo transfer (ET), and intracytoplasmic sperm injection (ICSI). We analyzed the semen parameters of 61 cycles in infertile couples undergoing IVF-ET and ICSI and determined DFI by sperm chromatin dispersion testing. Based on DFI, the patients were differentiated into a control group (DFI < 25%, *n* = 35) and a test group (DFI ≥ 25%, *n* = 26). Flow cytometry and immunofluorescence were used to investigate the extent of sperm reactive oxygen species (ROS) and apoptosis. We also investigated the effect of DFI on pregnancy outcomes of IVF-ET/ICSI. DFI was negatively related to sperm motility and positively correlated with ROS and apoptosis (*P* < 0.05). Abnormally elevated DFI reduced the rate of transplantable, high-quality embryos, implantation, clinical pregnancy, delivery, and live birth after IVF-ET, and increased the chance of early abortion per transfer cycle (*P* < 0.05). However, there was no significant correlation between DFI and fertilization rate, cleavage rate, transplantable rate, high-quality embryo rate, implantation rate, clinical pregnancy rate, early abortion rate, delivery rate and live birth rate when assisted by ICSI (*P* > 0.05). Sperm DNA integrity is crucial for fertilization and the development of healthy offspring. ROS may increase the level of DFI by inducing apoptosis in sperm.

## 1 Introduction

Globally, it is now estimated that infertility occurs in 7–15% of couples of reproductive age [1]. Infertility is defined as the failure of a couple to achieve a clinical pregnancy after 1 year of regular and unprotected sexual intercourse [2]. Female factors cause a large proportion of all infertility cases [3]. However, male infertility has increased over the last few decades and now accounts for 20–70% of infertile cases globally [4]. Furthermore, sole male factors are known to be responsible for 20–30% of cases and contribute to infertility in approximately 50% of cases, partly due to a general decline in sperm quality [2,5]. Routine semen analysis is the normal means of detecting sperm quality. However, individuals with semen analysis results within the normal range may still be unable to conceive, while individuals with abnormal results may still be able to conceive [6]. Consequently, semen analysis may be a poor predictor for the outcome of assisted reproductive technology (ART) [7].

DNA fragmentation index (DFI) assays could provide a more definitive evaluation of overall fertility status than routine semen analysis [8,9]. Different methodologies may detect different aspects of sperm DNA fragmentation (SDF). Sperm chromatin dispersion (SCD) and sperm chromatin structure assay (SCSA) may detect some aspects related to chromatin fragmentation, while the comet and terminal deoxynucleotidyl transferase (TdT)-mediated Dutp nick end labeling (TUNEL) assays could detect DNA breaks directly. The alkaline comet assay, the SCD test, SCSA, and the TUNEL assay are useful in that they can distinguish fertile and infertile patients [10]. A range of endogenous and exogenous factors can cause SDF and increased the DFI in sperm [11]. Dorostghoal et al. have shown that breaks in the sperm genome extending beyond a certain threshold can negatively affect fertility for both natural conception and ART [12]. DFI values are known to be elevated in physiological and/or pathological conditions such as inflammation, aging, certain infections, and following exposure to certain environmental factors [11,13].

Published evidence supports a negative association between sperm DNA damage and conventional IVF treatments, which can significantly reduce implantation and pregnancy rates [14]. There is controversy concerning the potential correlation between DFI and the outcomes of *in vitro* fertilization and embryo transfer/intracytoplasmic sperm injection (IVF-ET/ICSI) [12,15,16]. Unfortunately, previous research tended to investigate the relationship between the DFI and key sperm parameters. Here, we investigated the specific relationships between DFI and sperm motility, concentration, progressive rate (PR), morphology, reactive oxygen species (ROS), and apoptosis. Furthermore, we evaluated the effect of DFI on the key outcomes of IVF-ET/ICSI, including fertilization, embryo quality, and implantation to guide clinical intervention.

## 2 Materials and methods

### 2.1 Patient selection

This prospective research project was conducted at the Loudi Affiliated Hospital, Hengyang Medical School, University of South China, from August 2019 to April 2021. The ethics committee of Loudi Affiliated Hospital approved the study. Written and informed consent was obtained from all patients who volunteered to participate (reference: 2019-ethic review [science research]-044). In total, we included 61 cycles involving infertile couples who had attempted pregnancy by IVF-ET/ICSI. For all males, we used a kit to determine the level of SDF (the SCD test; Bred Life Science, Shenzhen, China) to determine the DFI. The DFI values were then used to classify the subjects into two groups: a control group (DFI < 25%) and a test group (DFI ≥ 25%). The following patients were included in this study: (1) patients with infertility caused by female pelvic cavity and tubal factors or male factors who met the IVF-ET/ICSI pregnancy aid indications; (2) women who were 25–40 years of age; (3) an antral follicle count (AFC) ≥5, a basal follicle stimulating hormone level ≤10 mIU/ml or an anti-mullerian hormone (AMH) level ≥1.2 ng/ml; (4) males who were 25–50 years of age; (5) both chromosomes, reproductive organs, and sexual function were normal. The following patients were excluded: (1) natural cycle pregnancy; (2) women with stage III to IV endometriosis; (3) males with azoospermia or undergoing thawing or testicular/epididymal aspiration; (4) males with testicular atrophy, genital tract malformation or urogenital infection; and (5) either the male or female had chromosomal abnormalities and other IVF-ET/ICSI pregnancy aid contraindications.


**Informed consent:** Informed consent has been obtained from all individuals included in this study.
**Ethical approval:** The research related to human use has complied with all the relevant national regulations and institutional policies, and is in accordance with the tenets of the Helsinki Declaration, and has been approved by the ethics committee of Loudi Affiliated Hospital.

### 2.2 General clinical data collection

We recorded the ages of the males and females who met the inclusion and exclusion criteria in this study. The duration of gonadotropin (Gn) use and the total amount of Gn used for ovulation induction were also recorded. The number of oocytes retrieved was recorded on the same day as oocyte retrieval.

### 2.3 Analysis of semen parameters

Semen samples were collected and processed in accordance with the standards described in the WHO Laboratory Manual for the Examination and Processing of Human Semen, Edition 5 (WHO, 5th edition) [17]. Routine sperm parameters were analyzed by computer-assisted semen analysis (CASA System, Spain). Prior to surgery, the slides and cover glasses were preheated, and the sperm counting plate was viewed with a ×10 objective lens. The light intensity was adjusted such that the system could automatically analyze at least 600 sperm and five fields of vision. After use, the system was cleaned with deionized ultrapure water and dried with absorbent paper. Next, we recorded several key parameters, including volume, concentration, motility, and PR. Sperm were stained with a Diff-Quik staining solution (Bred Life Science, Shenzhen, China) to facilitate morphological investigation and to determine the proportion of sperm with normal morphology (%).

### 2.4 Analysis of sperm DFI

The SCD test (Bred Life Science, Shenzhen, China) was used to assess males' most recent sperm DFI before the female ovulation induction therapy. Before testing, the room temperature was adjusted to 20–28°C. The easy gel tube was incubated at 80°C for 20 min and completely melted in a 35°C incubator. Then, the liquefied sperm concentration was adjusted to (5–10) × 10^6^/m1. Then, we took 60 µl of each sample to be tested with a sperm concentration of (5–10) × 10^6^/m1. The melted gel tube was added, mixed, and incubated at 35°C. The coated slides were pre-cooled in a 2–8°C refrigerator for 5 min and removed. Then, 30 µl of the sperm suspension prepared in step 1 was quickly added to the coated area of the slide. The sample was quickly covered with a cover glass (avoiding air bubbles) and placed in a 2–8°C refrigerator for 5 min to solidify. Subsequently, the slides were removed from the refrigerator, immediately immersed vertically into a reaction tank with reaction solution A, and incubated at 20–28°C for 7 min. Then, the slides were removed, and the residual liquid on the back and side edge of each slide was removed with filter paper. Then, the slide was immediately immersed vertically into the reaction tank with reaction solution B and incubated at 20–28°C for 25 min. Next, the slide was removed, and the residual liquid on the back and side of the slide was removed with filter paper. Then, the slide was immersed horizontally into a large amount of purified water for 5 min; the water was changed 1–2 times. Then, the slide was removed, and the residual liquid on the back and side edge of the slide was removed with filter paper. Then, the slide was immersed vertically in a reaction tank with 70% ethanol for 2 min. Then, the slide was removed, and the residual liquid on the back and side edge of the slide was removed with filter paper. The slide was immediately immersed vertically in a reaction tank with 90% ethanol for 2 min. Then, the slide was removed, and the residual liquid on the back and side of the slide was removed with a filter paper. Next, the slide was immersed vertically into a reaction tank with 100% ethanol for 2 min. Slides were then dried naturally in air. Next, each slide was covered with 15–20 drops of Swiss dye solution; this was followed by 30–40 drops of Swiss buffer as a wash step. Then, the stained specimens were rinsed with running water at room temperature for 15 min and then allowed to dry naturally in air. Then, we observed 500 sperm and quantified the number of sperm with DNA fragmentation. A distinct “halo” (dispersed DNA loops) was observed after removal of DNA-linked proteins; small halos, or the complete lack of halos, indicated DNA damage. If the radius of the halo was less than one-third of the minimum diameter of the sperm head, or no halo was formed, then the sperm was considered as one containing SDF, using the following formula: DFI (%) = number of sperm with DNA fragments/total number of observed sperm × 100% (normal reference value: DFI < 25%).

### 2.5 Semen sample collection and preparation

On the day of oocyte retrieval, males provided a sample of semen by masturbating directly into sterile plastic containers after 2–7 days of abstinence. Semen samples were processed in accordance with the recommendations given by the WHO. Semen samples were first liquefied at 37°C for 30 min. A Makler counting cell (Sefi-Makler, Israel) was then used to determine the sperm concentration. In addition, 0.5–1.0 ml of semen was mixed with a sperm freezing medium (Quinn’s, ART-8020, SAGE, USA) in a 1:1 ratio and stored in liquid nitrogen (at −196°C) for the subsequent detection of apoptosis and ROS. After semen liquefaction, 0.5 ml of 40% Pure Ception (Quinn’s, ART-2024, SAGE, USA) was taken with a pap straw and placed into a Falcon 2097 centrifuge tube. Then, 0.5 ml of 80% Pure Ception and 0.5 ml of 40% Pure Ception (Quinn’s, ART-2024, SAGE, USA) were added slowly to avoid air bubbles to create stratification. The liquefied semen was gently added to the density gradient medium and centrifuged at 300*g* for 20 min. The upper 40% Pure Ception and seminal plasma were then aspirated with a pap straw and discarded. The sperm precipitation at the bottom of the 80% Pure Ception liquid layer was then aspirated with another pap straw. This was re-suspended in 1.5 ml of the sperm washing medium and centrifuged at 300*g* for 7 min. The upper layer was cleaned with a pap straw, re-suspended in 1.5 ml of ART-1020-SPS medium and centrifuged at 300*g* for 5 min. The supernatant was discarded while the precipitate was retained, and resuspended with ART-1020-SPS medium to a volume of approximately 0.3 ml. Then, 10 μl of semen was used for routine analysis. The sperm suspension was slowly added to the lower layer of the equilibrated ART-1020-SPS culture medium. The tube was tilted to 45° and then incubated in an incubator at 35°C with 5% CO_2_ to await insemination.

### 2.6 Measurement of ROS in semen

ROS levels were determined using a Reactive Oxygen Species Assay Kit (E004, Nanjing Jiancheng Bioengineering Institute, China). The frozen semen was thawed at 37°C in a constant temperature water bath for 3–5 min, and washed twice (1,000*g* × 5 min) in 0.9% normal saline (NS). A 2,7-dichlorofuorescin diacetate (DCFH-DA) probe was diluted (1:1,000) with 0.9% NS to a final concentration of 10 μM and then used to suspend sperm cells to a concentration of (1–2) × 10^7^/ml. Following incubation in the dark for 45 min (mixed upside down every 5 min to make full contact with the cells), the suspension was centrifuged and washed twice (1,000*g* × 5 min) in 0.9% NS. The sperm were then resuspended in 0.9% NS and analyzed by flow cytometry (BD FACS Calibur, Becton Dickinson, San Jose, CA, USA) and fluorescence microscopy (EVOS M7000 3D, Thermo, USA). In each group, ROS was expressed as the mean fluorescence intensity.

### 2.7 Assessment of apoptosis in semen

Apoptosis was assessed using the AnnexinV-FITC/PI Double Staining Apoptosis Detection kit (G003; Nanjing Jiancheng Bioengineering Institute, China). The frozen semen was thawed at 37°C constant temperature water bath for 3–5 min. An aliquot of semen ([1–2] × 10^6^/ml sperm) was washed twice (1,000*g* × 5 min) in 0.9% NS and the supernatant was discarded. Then, 500 μl of AnnexinV-FITC conjugate and 5 μl of AnnexinV-FITC were added. Next, we added 5 μl of propidium iodide (PI) solution. Following incubation in the dark for 10 min, the suspension was analyzed by flow cytometry (BD FACS Calibur; Becton Dickinson, San Jose, CA, USA) and fluorescence microscopy (EVOS M7000 3D; Thermo, USA). Then, the sperm apoptosis rate (%) = number of apoptotic sperm/total number of observed sperm.

### 2.8 Conditions and parameters were measured by flow cytometry

A FACS Canto II flow cytometer from Becton-Dickinson (excitation light source, 450 mW argon ion laser, laser wavelength, 488 nm, emission wavelength Em = 530 nm) was used. Signals from FITC and DCFH were collected with the FL1 channel and PI signals were collected with FL2 and analyzed using BD FACSDiva 8.0.1 software. The forward scattering (FSC) parameter was set a threshold of 800 to control the injection velocity at 500 s/s. The FSC of the sperm is the abscissa, the side scatter (SSC) is the ordinate, and the sample with normal DNA fragments is used as the control. FSC and SSC values were adjusted, the target cell population was selected in the central position of the collection box, and then FL1 and FL2 compensation values were adjusted. The normal cells treated without apoptosis induction were used, and control fluorescence compensation was used to remove spectral overlap, and set the two-parameter dot map of the fluorescence signal. FITC PI^−^ indicated double negative cells, FITC^+^ PI^−^ the apoptotic cells, DCFH-DA^−^ the negative cells and DCFH-DA^+^ the ROS sperm. The control apoptosis rate was adjusted below 5.0%, and 20,000 cells were collected per sample, and samples were collected one by one.

### 2.9 Controlled ovulation induction and oocyte collection

Sixty-one female patients underwent controlled ovarian stimulation to achieve an appropriate number of oocytes. When there were 2–3 dominant follicles with a diameter ≥ 17–18 mm and the blood estrogen level reached 200–300 pg/ml for each dominant follicle (≥14 mm), we injected human chorionic gonadotropin, and B-ultrasound was performed 34–38 h later for oocyte collection. The oocytes were incubated at 37°C, and 5% CO_2_ for later use.

### 2.10 Assessment of IVF-ET/ICSI outcomes

Oocytes were cultured for 2–4 h and inseminated. Fertilization status was observed 16–18 h after insemination; we recorded fertilization and cleavage embryos. Cleavage status was observed 42–44 h after insemination, and the number of transplantable and high-quality embryos was determined. Transplantable embryos refer to embryos from two pronuclear embryos with a number of blastomeres ≥5 cells, with uniform or uneven blastomeres, and the proportion of embryonic fragments < 30% when observed 66–68 h after IVF or ICSI. Embryo evaluation was performed 66–68 h after insemination. We then performed blastocyst culture and ET as appropriate. Subsequently, we recorded blastocyst formation, implantation, clinical pregnancy, early abortion, and delivery or live birth in each IVF-ET/ICSI treatment cycle. Patients were tested for serum b-hCG assay 12–14 days post-ET. Implantation and clinical pregnancy were defined as the presence of a gestational sac confirmed by B-ultrasound 26–28 days after ET.

### 2.11 Statistical analysis

SPSS version 21.0 (IBM, Chicago, IL, USA) was used for all statistical analyses. Normality was evaluated using the Shapiro–Wilk test. Measurement data are expressed as mean ± standard deviation, and comparisons between the two groups were carried out with Student’s *t*-test for independent samples. Count data were compared between groups by Pearson’s *χ* ^2^ test. Pearson correlation analysis was used to identify potential correlations. *P* < 0.05 was considered statistically significant.

## 3 Results

### 3.1 Basic clinical information

There were no significant differences between the two groups in terms of BMI, age, duration of Gn, dosage of Gn, and number of oocytes retrieved (*P* > 0.05; Table 1).

**Table 1:** Comparison of patient characteristics between the two groups

Group	Control group (DFI < 25%) (mean ± SD)	Test group (DFI ≥ 25%) (mean ± SD)	*F*	*t*
Cycle (numbers)	35	26	—	—
Female age (years)	34.46 ± 3.12	33.85 ± 3.79	4.418	0.043
Male age (years)	36.71 ± 4.64	36.65 ± 5.95	2.967	0.690
Female BMI (kg/m^2^)	23.11 ± 2.67	24.28 ± 3.02	0.737	−1.596
Male BMI (kg/m^2^)	24.35 ± 3.40	24.77 ± 3.46	0.015	−0.476
Duration of Gn used (days)	10.23 ± 1.63	10.38 ± 1.53	0.034	−0.380
Dosage of Gn used (IU)	2568.93 ± 820.42	2824.52 ± 679.43	0.962	−1.292
Oocytes retrieved (numbers)	10.80 ± 4.26	11.42 ± 3.69	1.794	−0.598

^*^ *P* < 0.05, ^**^ *P* < 0.01, compared with the control group; BMI, body mass index; Gn, gonadotropin.

### 3.2 Semen parameters

There was no significant difference between the two groups regarding semen volume, concentration, and normal morphology (*P* > 0.05). However, sperm motility and PR were significantly different between the two groups (*P <* 0.05; Table 2).

**Table 2:** Comparison of semen parameters between the two groups

Group	Control group (DFI < 25%) (mean ± SD)	Test group (DFI ≥ 25%) (mean ± SD)	*F*	*t*
Cycle (numbers)	35	26	—	—
Volume (ml)	2.69 ± 0.82	2.65 ± 0.88	0.022	0.145
Concentration (10^6^/ml)	98.09 ± 63.11	80.65 ± 51.24	0.067	1.153
Motility (%)	50.43 ± 9.48	42.92 ± 11.42^**^	0.270	2.802
PR (%)	36.34 ± 8.34	31.08 ± 9.94^*^	0.651	2.247
Normal morphology (%)	3.50 ± 1.58	3.20 ± 1.70	0.295	0.709

^*^ *P* < 0.05, ^**^ *P* < 0.01, compared with the control group; PR, progressive rate.

### 3.3 Levels of ROS

**Figure 1:**
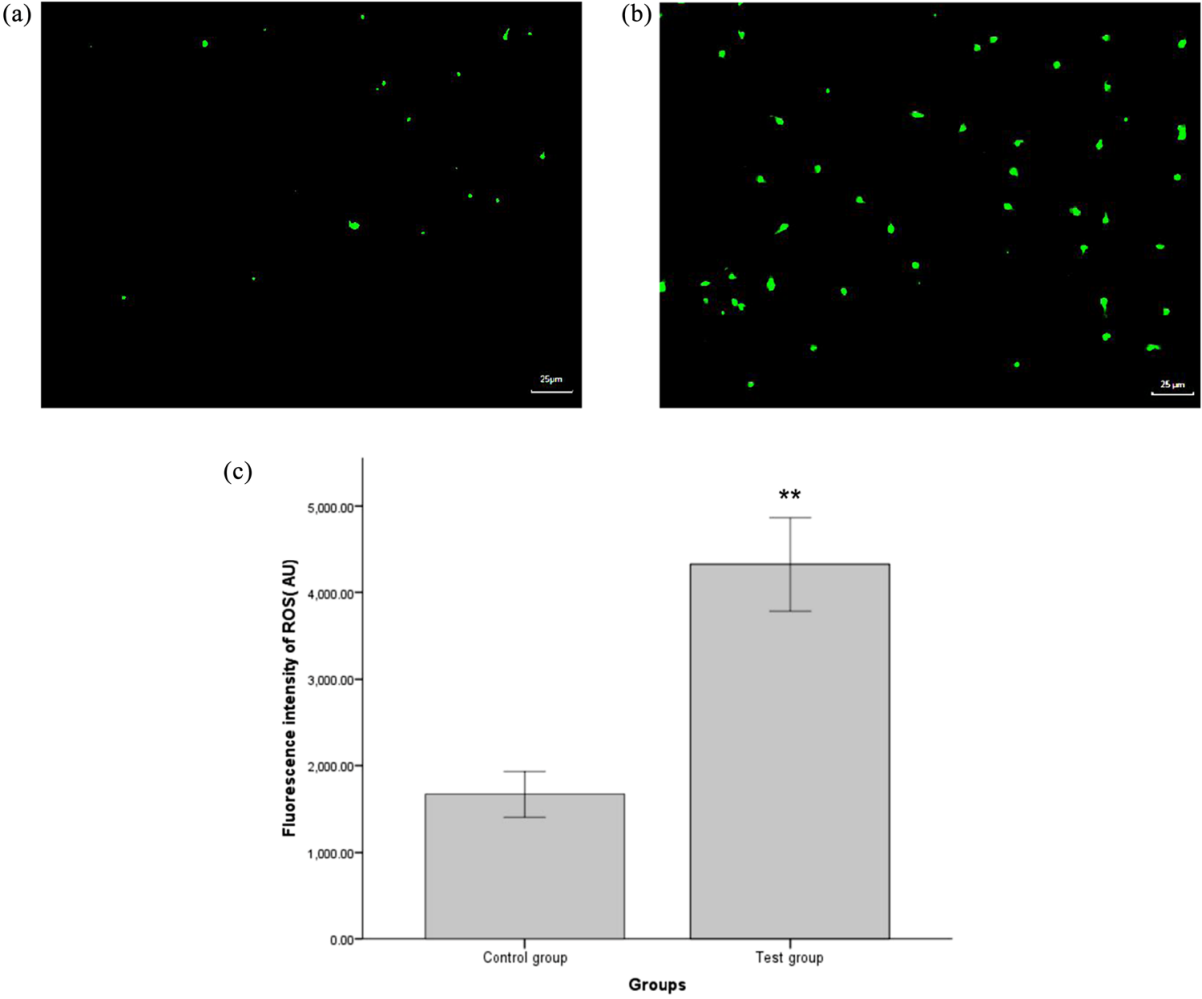
ROS levels of sperm in the control group (a) and test groups (b). ROS production was detected by fluorescence microscopy after DCFH-DA staining; (a and b) (×400). Green staining indicated ROS-positive cells. The fluorescence intensity of ROS in the test group was significantly higher than that in the control group (c). ^*^ *P* < 0.05, ^**^ *P* < 0.01, compared with the control group.

The majority of sperm in the test group exhibited positive green fluorescence (Figure 1b); however, such fluorescence was rarely seen in sperm from the control group (Figure 1a). Flow cytometry showed that the fluorescence intensity of ROS in the control group was 1670.00 ± 766.26 AU (arbitrary unit); this was significantly higher than that in the test group 4323.88 ± 1338.04 AU (Figure 1c, *P* < 0.05).

### 3.4 Comparison of apoptosis between groups

**Figure 2:**
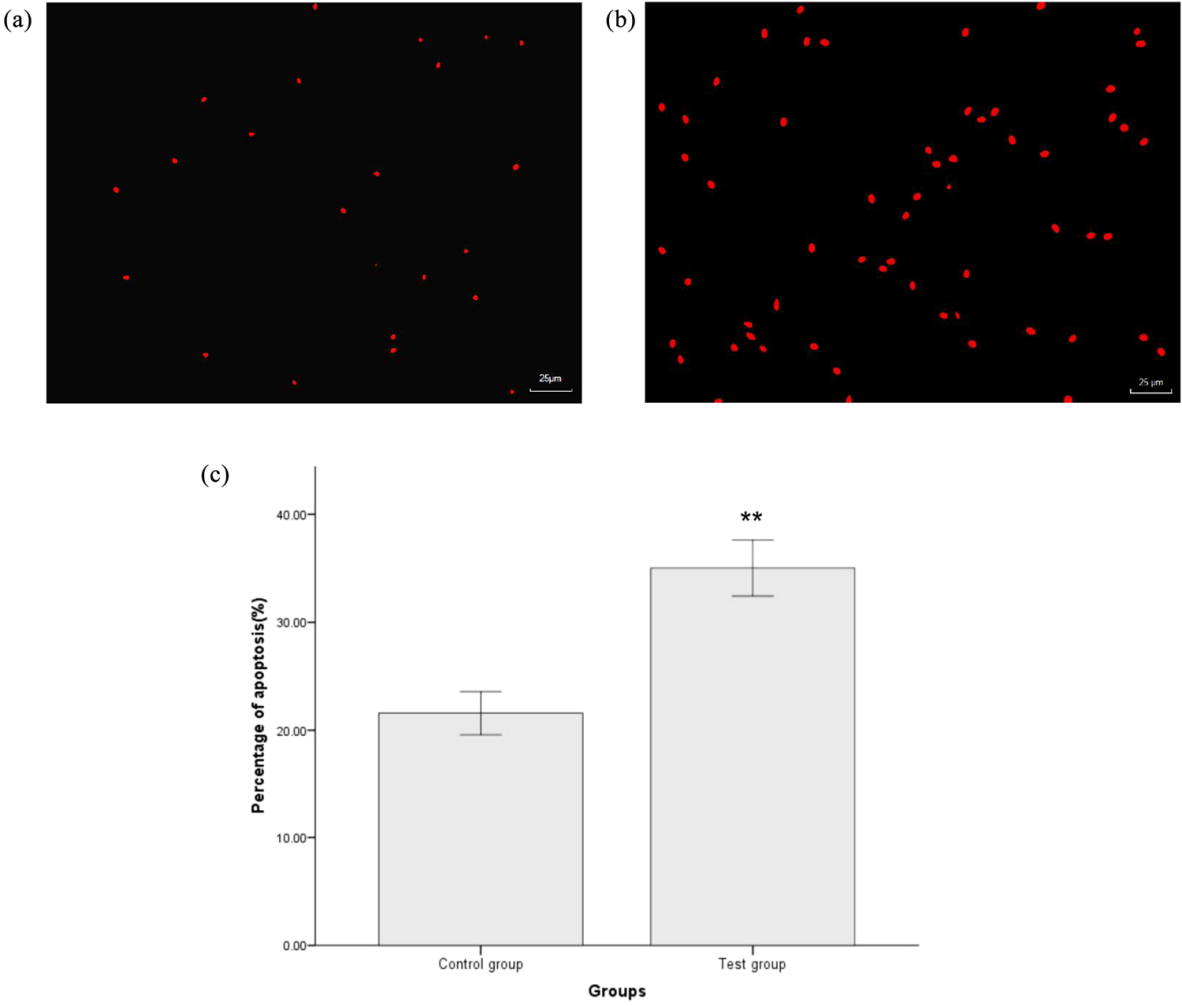
Apoptosis levels of sperm in the control group (a) and test groups (b). ROS production was detected by fluorescence microscopy after AnnexinV-FITC/PI staining; (a and b) (×400). Red staining indicated ROS-positive cells. The percentage of apoptosis in the test group was significantly higher than that in the control group (c). ^*^ *P* < 0.05, ^**^ *P* < 0.01, compared with the control group.

The majority of sperm in the test group exhibited positive red fluorescence; however, this was rarely seen in sperm from the control group (Figure 2a and b). Flow cytometry showed that the percentage of apoptosis in the control group was 21.58 ± 5.82%; significantly higher than that in the test group (35.02 ± 6.41%; Figure 2c, *P* < 0.05).

### 3.5 Correlation between DFI and male age, BMI, semen parameters, ROS, and apoptosis

**Figure 3:**
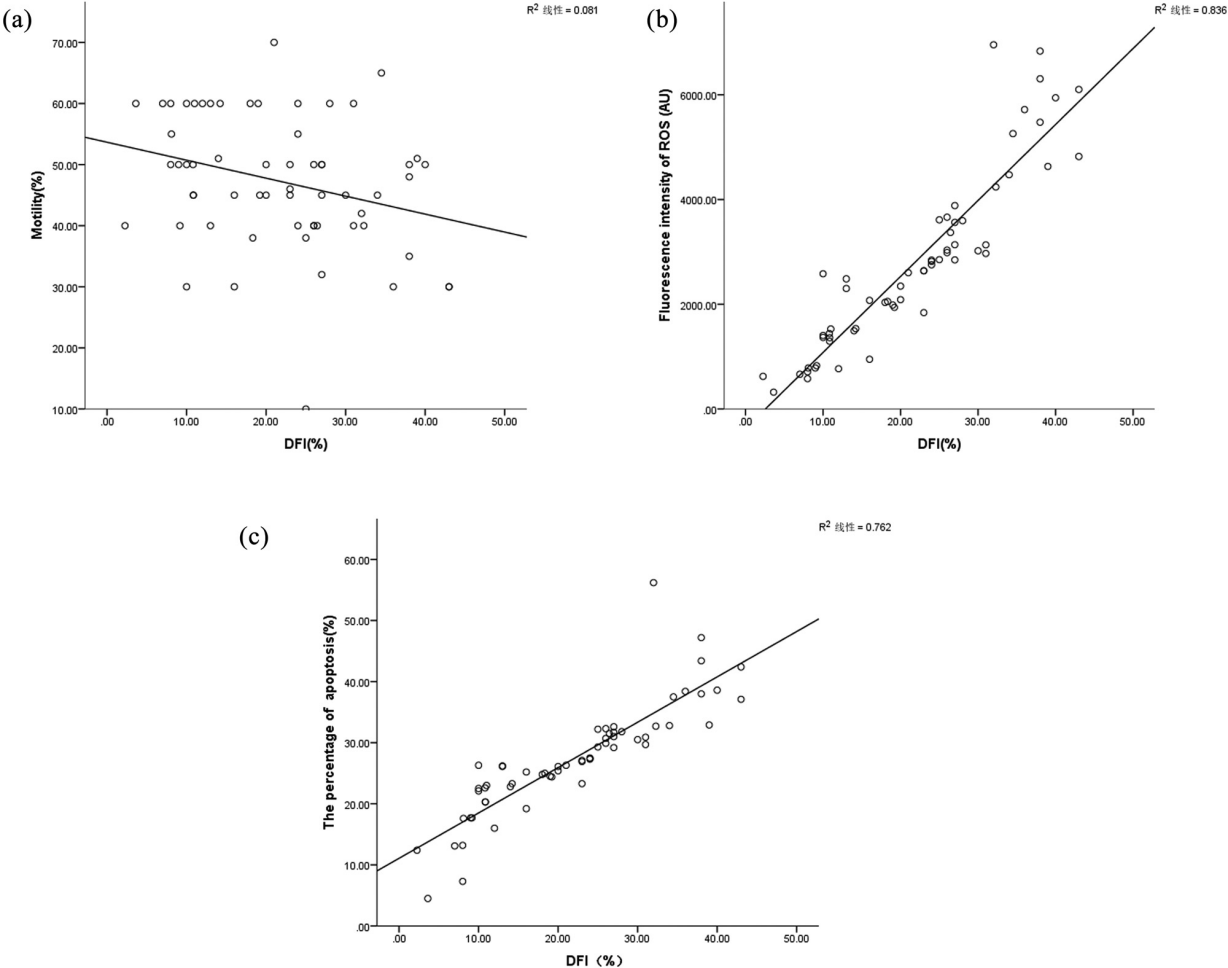
Relationship between DFI and sperm motility, apoptosis rate, and ROS. (a) Correlation between DFI and sperm motility (*r* = −0.263, *P* < 0.05). (b) Correlation between DFI and ROS (*r* = 0.900, *P* < 0.05). (c) Correlation between DFI and sperm apoptosis rate (*r* = 0.864, *P* < 0.05).

There were no correlations between DFI and male age, BMI, semen volume, sperm concentration, PR, and normal morphology rate (*P* > 0.05). However, there was a negative correlation between DFI and semen motility, and a highly positive correlation between DFI and both apoptosis and ROS (*P <* 0.05; Table 3, Figure 3).

**Table 3:** Correlations between DFI and male age, BMI, semen parameters, ROS, and apoptosis

	*r*	*P*
Male age (years)	0.065	0.617
Male BMI (kg/m^2^)	0.051	0.695
Volume (ml)	−0.198	0.126
Concentration (10^6^/ml)	−0.124	0.341
Motility (%)	−0.285^*^	0.026
PR (%)	−0.210	0.104
Normal morphology (%)	−0.083	0.526
ROS (AU)	0.914^**^	0.000
Apoptosis rate (%)	0.873^**^	0.000

^*^ *P* < 0.05, ^**^ *P* < 0.01; BMI, body mass index; PR, progressive rate; AU, arbitrary unit.

### 3.6 IVF-ET outcomes

There was no significant difference between the two groups regarding fertilization rate and cleavage rate (*P* > 0.05). However, the transplantable embryo rate, high-quality embryo rate, implantation rate, clinical pregnancy rate, early abortion rate, delivery rate, and live birth rate were significantly different when compared between the two groups (*P* < 0.05, Table 4). Furthermore, no stillbirth, stillbirth, malformed fetuses, or neonatal birth defects occurred in either of the two groups.

**Table 4:** Comparison of IVF-ET outcomes between the two groups

Group	Control group (DFI < 25%)	Test group (DFI ≥ 25%)	*χ* ^*2*^
Cycle (number)	28	16	—
Fertilization rate (%)	81.61 (244/299)	77.65 (132/170)	1.068
Cleavage rate (%)	97.95 (239/244)	99.24 (131/132)	0.273
Transplantable embryo rate (%)	87.87 (210/239)	74.05 (97/131)^**^	11.440
High-quality embryo rate (%)	48.26 (97/201)	30.70 (35/114)^**^	9.211
Implantation rate (%)	53.06 (26/49)	27.59 (8/29)^*^	4.808
Clinical pregnancy rate (%)	78.57 (22/28)	37.50 (6/16)^**^	7.422
Early abortion rate (%)	4.55 (1/22)	50.00 (3/6)^*^	—
Delivery rate (%)	75.00 (21/28)	18.75 (3/16)^**^	12.994
Live birth rate (%)	75.00 (21/28)	18.75 (3/16)^**^	12.994

^*^ *P* < 0.05, ^**^ *P* < 0.01, compared with the control group; positive number/total number in parentheses.

### 3.7 ICSI outcomes

There was no significant correlation between DFI and the rate of fertilization, cleavage, transplantable, high-quality embryos, implantation, clinical pregnancy, early abortion, delivery, and live birth when assisted by ICSI (*P* > 0.05, Table 5). However, we found that the fertilization rate, transplantable rate, high-quality embryos rate, implantation rate, clinical pregnancy rate, delivery rate, and live birth rate in the test group were lower than those in the control group. There was no incidence of stillbirth, malformed fetuses, or neonatal birth defects in either of the two groups.

**Table 5:** Comparison of ICSI outcomes between the two groups

Group	Control group (DFI < 25%)	Test group (DFI ≥ 25%)	*χ* ^*2*^
Cycle (number)	7	10	—
Fertilization rate (%)	92.86 (65/70)	86.49 (96/111)	1.773
Cleavage rate (%)	98.46 (64/65)	100.00 (96/96)	0.039
Transplantable embryo rate (%)	82.81 (53/64)	72.28 (73/101)	2.409
High-quality embryo rate (%)	37.70 (23/61)	25.27 (23/91)	2.674
Implantation rate (%)	46.15 (6/13)	30.00 (6/20)	—
Clinical pregnancy rate (%)	71.43 (5/7)	60.00 (6/10)	—
Early abortion rate (%)	0.00 (0/5)	16.67 (1/6)	—
Delivery rate (%)	71.43 (5/7)	50.00 (5/10)	—
Live birth rate (%)	71.43 (5/7)	50.00 (5/10)	—

^*^ *P* < 0.05, ^**^ *P* < 0.01, compared with the control group; positive number/total number in parentheses.

## 4 Discussion

In this study, we observed significant differences in sperm motility and PR when compared between the two groups; these findings were similar to those of Khalafalla et al. [18]. We also observed a negative association between DFI and sperm motility. Excessive levels of ROS may induce lipid peroxidation of the sperm membrane, reduce the mitochondrial membrane potential and the production of ATP, and reduce the motility of sperm [19]. However, we failed to detect a correlation between DFI and male BMI. Thus, we need to expand the sample size in the future and group samples according to age.

It has been reported that the excessive production of ROS and the reduced levels of antioxidants in semen may play a vital role in DNA damage and apoptosis, and they were more oxidized in infertile males than in fertile males [20,21,22]. This prospective study showed that the levels of ROS and apoptosis increased significantly when the sperm DFI increased abnormally, apparent as a significant linear relationship. In certain circumstances, the stability of the mitochondrial electron transport chain protein and transcription system can be reduced, thus inducing cytochrome C and apoptosis inducing factor in the mitochondria to be transported to the mitochondrial membrane, further forming apoptotic factors and degrading DNA; this may even cause the irreversible arrest of spermatogenesis when excessive levels of ROS are produced [23,24,25]. In the later stages of sperm development, DNA repair proteins downstream of the base excision repair pathway are lacking. Thus, ROS can influence base modification, thus inducing single- or double-strand DNA breaks and destroying the integrity of sperm DNA [26].

Sperm DFI cannot be used to predict embryo quality or pregnancy rate following IVF-ET/ICSI [15,27,28]. Alvarez Sedó et al. previously reported that sperm DFI was negatively associated with pregnancy rates [16]. We observed that abnormally elevated DFI values reduced the rates of transplantable embryos, high-quality embryos, implantation, clinical pregnancy, delivery and live birth after IVF-ET and increased the risk of early abortion per transfer cycle. Furthermore, the transplantable embryo rate, high-quality embryo rate, implantation rate, clinical pregnancy rate, delivery rate, and live birth rate were all reduced in ICSI treatment as the DFI values increased. The factors underlying these findings may be related to the following factors. First, the fertilization process may be regulated by maternal genes [29]. However, the sperm DNA damage may cause different degrees of functional defects in sperm, which may affect the developmental potential of zygotes [30]. Second, embryonic genome activation mostly occurs in the 4–8 cell stage. The sperm head nucleus carries paternal genes that begin to undergo activation; toxic metabolites on the sperm membrane may affect embryo development and the uterine environment may lose synchronization, eventually reducing the probability of clinical pregnancy and increasing the risk of early abortion [31]. Moreover, until the middle and third trimester of pregnancy, the utero-placental circulation is established; the estrogen and progesterone secreted mainly through the placenta and trophoblast layer continue to maintain gestation [32].

DNA damage has been proposed as a mechanism change for DNA methylation that may significantly impact transcriptional regulation [33,34,35,36]. If oxidization occurs in regions that should not be associated with the demethylation process post-fertilization, it may interfere with the developmental processes associated with the rearrangement of the sperm chromatin [37]. These processes can increase the risk of poor embryonic quality and implantation failure and miscarriage rate [38,39]. Consequently, we suspect that excessive levels of ROS may increase apoptotic-like changes in sperm that mediate the cell cycle and programmed cell death, thus resulting in SD; this affects fertility and has an adverse effect on pregnancy outcome. However, we did not separate the effects of IVF-ET and ICSI in this study; this needs to be investigated in future research with a control group of couples with normal fertility.

## 5 Conclusion

With these data, it can be concluded that the SCD test is useful to distinguish fertile and infertile patients. Sperm DNA integrity is crucial for fertilization and the development of healthy offspring. ROS may increase the level of DFI by inducing apoptosis in sperm.
